# The AST/ALT Ratio (De Ritis Ratio) Represents an Unfavorable Prognosis in Patients in Early-Stage SFTS: An Observational Cohort Study

**DOI:** 10.3389/fcimb.2022.725642

**Published:** 2022-02-08

**Authors:** Lianzi Wang, Yang Xu, Shubing Zhang, Asma Bibi, Yuanhong Xu, Tao Li

**Affiliations:** Department of Clinical Laboratory, The First Affiliated Hospital of Anhui Medical University, Hefei, China

**Keywords:** SFTS, AST/ALT-ratio, De Ritis ratio, prognosis, clinical characteristics

## Abstract

**Background:**

Severe fever with thrombocytopenia syndrome (SFTS), a widely prevalent infectious disease caused by severe fever with thrombocytopenia syndrome virus (SFTSV) that carries with it a high mortality rate, has emerged to be a public health concern. This study aimed to investigate the epidemiological and clinical characteristics of patients infected with SFTSV, seeking novel prognostic risk factors for SFTS.

**Methods:**

In this retrospective and cross-sectional study, confirmed SFTS patients from the First Affiliated Hospital of Anhui Medical University were enrolled from September 1, 2019, to December 12, 2020. Cases were analyzed for epidemiological, demographic, clinical, and laboratory data. Logistic regression models were used to assess the association between predictors and outcome variables. A generalized additive mixed model (GAMM) was conducted to analyze the trending shift of aspartate aminotransferase/alanine transaminase-ratio (AST/ALT-ratio) and platelet (PLT) in SFTS patients treated with ribavirin. p values ≤ 0.05 were considered statistically significant.

**Results:**

Clinical and laboratory results of 107 hospitalized patients with SFTSV infection were retrospectively described. The mean age at onset of disease was 60.38 ± 11.29 years old and the ratio between male and female was 1:1.2. Fever and thrombocytopenia are hallmark features of SFTS. Furthermore, multiple cases also experienced neurological complications, gastrointestinal/skeletal muscle symptoms together with other non-specific clinical manifestations; laboratory dataset outcomes reported dysregulated levels for routine blood biomarkers, coagulation function, and biochemistry. Overall, 107 patients were segregated into two groups according to patient condition at the clinical endpoint (survivors/non-survivors). SFTS survivors had a higher level of PLT- counts, total protein (TP), and estimated glomerular filtration rate (eGFR), while levels of activated partial thromboplastin time (APTT), thrombin time (TT), D-dimer (D-D), fibrinogen degradation products (FDP), ALT, AST, AST/ALT-ratio, creatinine (Cr), creatine phosphokinase (CK) and procalcitonin (PCT) was higher in non-survivors. Results from univariate Cox regression revealed that elevated levels of FDP, TT, AST/ALT-ratio, PCT, as well as decreased eGFR level and presence of central nervous system symptoms (CNS), were significant predictors for SFTS prognostic, results from multivariate logistic regression analysis in three adjusted models showed AST/ALT-ratio and PCT were independent risk factors for the prognosis of SFTS patients. Kaplan–Meier survival analysis showed that SFTS patients with AST/ALT-ratio >2.683 were associated with a shorter futime (means survival time), therefore indicating an unfavorable prognosis. Treatment with ribavirin could increase PLT count while decreasing AST/ALT-ratio within SFTS patients.

**Conclusion:**

SFTS is an emerging infectious disease, possibly leading to multiple-organ injury; AST/ALT-ratio was an independent risk factor for the prognosis of SFTS patients. Further investigation should be performed in order to gain more knowledge on this disease and guide clinical management.

## Background

Severe fever with thrombocytopenia syndrome (SFTS) is a tick-borne disease first identified in China in 2009, that rapidly spread to other provinces in central, eastern, and northeastern regions (Yu et al., 2011) no longer restrained to China, SFTS patients were also diagnosed in Japan and Korea in 2012, followed by rapid spread into the United States and other countries (Liu et al., 2014; Saijo, 2018). The initial case mortality rate of SFTS reached up to 30% in the early years, though the estimated mortality rate has decreased to 12% in China while a high fatality rate was still reported in Japan and Korea (Robles et al., 2018). Humans are the main host for SFTSV, people become infected mainly *via* tick-bites, then lead to human-to-human transmission, apart from humans, other mammals and rodents also could be SFTSV hosts (Lee et al., 2019; Chung et al., 2020), direct contact with the body fluids of infected animals leads to SFTSV infections in humans as well (Kobayashi et al., 2020). A recent study detected SFTSV RNA in semen following its disappearance from serum presence, indicating a possible sexual transmission of SFTSV (Koga et al., 2019), a report pointed out that the formation of aerosols was also a potential transmission route of SFTSV (Moon et al., 2018).

Clinical manifestations of SFTS are non-specific, including fever, fatigue, muscular soreness, nausea, and vomiting, further easily leading to differential diagnoses of injury with multiple symptoms including digestive/neurological symptoms, musculoskeletal system among others. Fulminant myocarditis was recently first reported as a complication with SFTS (Miyamoto et al., 2019). Several patients progress rapidly into multiple organ dysfunction syndrome (MODS). Mainly, laboratory profiles of SFTS consisted of thrombocytopenia and leukocytopenia. SFTS is listed as a disease requiring urgent research and development by the World Health Organization (WHO) in 2017, there is no available effective SFTS treatment presently, antiviral drugs are typically utilized in the treatment of SFTS. In addition, steroid pulse therapy and plasma exchange were included in proposed treatments for SFTS in recent years (Choi et al., 2018; Nakamura et al., 2018; Takayama-Ito and Saijo, 2020), however, the effectiveness of all such treatments remains unclear, with mechanisms of disease pathogenesis still being unclear. Consequently, there is an urgent need to focus on SFTSV-infected patients.

This study aimed to investigate the epidemiological and clinical characteristics of patients infected with SFTSV, and also to identify predictive biomarkers for fatality, in order to provide interventional benefit in early-stage SFTS cases and avail the optimal clinical management strategies for such clinical scenarios.

## Patients and Methods

### Patients’ Enrollment and Data Collection

This was a retrospective follow-up study that included 107 hospitalized patients from the First Affiliated Hospital of Anhui Medical University, diagnosed with SFTS and recruited from September 1, 2019, to December 12, 2020. This research was approved by the Ethics Review Committee of the First Affiliated Hospital of Anhui Medical University and was presented in accordance with the principles of the declaration of Helsinki. All patients underwent a comprehensive physical examination at the time of admission, including blood routine, biochemical, etiological, and neurologic evaluation.

SFTS patients were defined according to results of the real-time reverse transcriptase-polymerase chain reaction (RT-PCR) or detecting IgM/IgG antibodies specific for SFTSV based on serology. Basial information of SFTS patients, including epidemiological, demographic, clinical, and laboratory data, was obtained from individual medical records. All patients were followed up for 60 days post-discharge.

### Blood Samples

SFTS patient blood samples were collected (in EDTA-, sodium citrate-, or nil-coagulant tubes) after fasting for at least 8 hours and immediately analyzed. Blood samples collected in EDTA were used for blood routine examination straight away; blood samples collected in sodium citrate were centrifuged for 15 minutes at 3500 r/s and employed for hemostatic examination while blood samples with no anticoagulation were centrifuged for 5 minutes at 3500 r/s for biochemical examination. Analyses were continuous, and the list of biochemical markers analyzed in this laboratory is listed in **Table 1**. The laboratory was certified by ISO 15189, with inter-/intra-batch coefficients of variation being both < 8%.

**Table 1:** Laboratory indexes and their abbreviation name.

Full name	Abbreviation name
Hemoglobin	Hb
White blood cell	WBC
platelet	PLT
Activated partial thromboplastin time	APTT
Prothrombin time	PT
Thrombin time	TT
Fibrinogen degradation products	FDP
D-dimer	D-D
γ-glutamyl transferase	GGT
Alkaline phosphatase	ALP
Alanine transaminase	ALT
Aspartate aminotransferase	AST
Lactate dehydrogenase	LDH
Total protein	TP
Albumin	ALB
Creatine phosphokinase	CK
Creatinine	Cr
Estimated glomerular filtration rate	eGFR
Uric Acid	UA
C-reactive protein	CRP
procalcitonin	PCT

### Statistical Analysis

All continuous variables were expressed as the mean ± standard deviation (SD) for normal distribution or median (interquartile range) for skewed distribution, while categorical variables were described as a frequency or percentage. In addition, one-way ANOVA (normal distribution), Kruskal-Wallis H (skewed distribution) test, and chi-square test (categorical variables) were employed to determine any significant differences between the means and proportions of the groups. Univariate and multivariate cox regression analyses were applied to identify factors associated with SFTS severity. However, variables with a p-value less than 0.1 in the univariate Cox regression were brought in multivariate Cox regression with a stepwise method, in order to determine independent prognosis factors for SFTS. Hazard ratios (HRs) estimated from the Cox analysis were reported as relative risks with 95% confidence intervals (CIs).

Receiver operating curve (ROC) analysis was used for the calculation of optimal cut-off value for AST/ALT-ratio. Kaplan–Meier curves were applied to perform the associations between AST/ALT-ratio and mortality. The relationship between AST/ALT-ratio and other clinical parameters was determined through paired Spearman’s correlation. Spline smoothing of the generalized additive models was performed to explore the relationship between AST/ALT-ratio and mortality.

In order to assess the shifting trend of AST/ALT-ratio between survivors and non-survivors, a generalized additive mixed model (GAMM) was conducted to implement this analysis. All models also included intercept and time as random terms. Random effects allow each participant’s beginning value to vary from the population average (intercept) and the longitudinal trajectory to vary from the population average longitudinal trajectory (slope), with statistically relevant (p < 0.1 in two-tailed t test or Mann-Whitney test) cases were included in the models.

In order to avoid the reduced efficiency and bias of statistical tests caused by the direct exclusion of missing values, we used multiple imputations (MI) to estimate missing values, a statistical approach based on a chained equation approach and generated 5 complete data for analysis.

Analyzes were performed using the SAS package (version 8.1, SAS Institute Inc., Cary, North Carolina) and SPSS 26.0. For all comparisons, a two-tailed with p < 0.05 was considered statistically significant.

## Results

### Demographics and Clinical Characteristics of SFTS Patients

A total of 107 patients diagnosed with SFTS were enrolled in our study, the average age of subjects was 60.38 ± 11.29 years old, ranging from 27 to 86 years old, the ratio between male and female was 1:1.2, a total of 32 cases were confirmed to have a tick-bite history prior to illness onset, a total of 10 patients (9.35%) were infected due to close contact with SFTS patients, with one case being an attending nurse who infected through direct contact with SFTS patient body fluid/s. The median time from admission to discharge was 10 days (interquartile range, 8 to 15). A total of 26 mortalities were registered, with MODS being the leading cause of death. However, although 46.15% (12/26) of patients died due to MODS, infection was also an important contributing factor to death (**Table 2**).

**Table 2:** Demographics and clinical characteristics of SFTS.

General characteristics	Total (n=107)
Age (years)	60.38 ± 11.29
<50	17 (15.89%)
50-70	70 (65.42%)
>70	20 (18.69%)
Gender	
Male	48 (44.86%)
female	59 (55.14%)
BMI (Kg/m^2^)	
Way of occupation	
Bite by ticks, n (%)	32 (29.91%)
Contact with SFTS patients, n (%)	10 (9.35%)
Smoking history, n (%)	12 (11.21%)
Drinking history, n (%)	19 (17.76%)
surgery, n (%)	15 (14.02%)
cardiovascular diseases, n (%)	24 (22.43%)
diabetes, n (%)	16 (14.95%)
complication, n (%)	38 (35.51%)
Hospitalization days	
<7	17 (15.89%)
7-14	60 (56.07%)
>14	30 (28.04%)
Time from onset to admission (days)	
<3	26 (24.30%)
4-7	64 (59.81%)
>7	17 (15.89%)
Highest body temperature	
<37.6	9 (8.41%)
37.6-38.6	31 (28.97%)
38.7-39.6	51 (47.66%)
>39.6	13 (12.15%)
Clinical outcome	
survivors, n (%)	81 (75.70%)
non-survivors, n (%)	26 (24.30%)
Cause of death	
MODS	12 (46.15%)
Viral encephalopathy	2 (7.69%)
Disseminated intravascular coagulation (DIC)	5 (19.23%)
Severe acute respiratory syndrome (SARS)	1 (3.85%)
Infection	6 (23.08%)

Symptoms of all investigated SFTS patients on admission were shown in **Table 3**. Fever was the first symptom upon illness onset in most patients (95/107), and the highest body temperature in patients was 38.77 ± 0.09. The symptom manifestation prevalence was followed by fatigue (70/107, 65.4%), rigor (66/107, 61.68%), cough (11/107, 10.28%), and in approximately 35.51% patients was accompanied by muscular soreness. In addition, 47.66% of patients suffered from gastrointestinal symptoms, including nausea, vomiting, diarrhea, poor appetite, and abdominal pain, a total of 18 (16.82%) patients presented dermatological issues, while 38 cases had severe neurological symptoms included dizziness, headache and loss consciousness. Furthermore, patients that experienced CNS symptoms had a higher mortality ratio.

**Table 3:** Symptomatic characteristics of patients with SFTSV infection at admission.

Symptom on admission	Total (107)	survivors (81)	non-survivors (26)	p
Fever	95 (88.79%)	72 (88.89)	23 (88.46%)	0.546
Fatigue	70 (65.42%)	53 (65.43%)	17 (65.38%)	0.996
Skin change	18 (16.82%)	13 (16.05%)	5 (19.23%)	0.706
Rigor	66 (61.68%)	52 (64.20%)	14 (53.85%)	0.345
CNS	38 (35.51%)	24 (29.63%)	14 (53.85%)	0.025
Diarrhea	40 (37.38%)	33 (40.74%)	7 (26.92%)	0.205
Vomiting	20 (18.69%)	17 (20.99%)	3 (11.54%)	0.282
Nausea	24 (22.43%)	19 (23.46%)	5 (19.23%)	0.653
muscular soreness	38 (35.51%)	27 (33.33%)	11 (42.31%)	0.405
Cough	11 (10.28%)	10 (12.35%)	1 (3.85%)	0.214

Furthermore, statistical analyses were performed on laboratory results of SFTS patients, including complete blood count, biochemical analysis, coagulation testing, and CRP/PCT evaluations (**Table 4**). A total of 94.39% (101/107) of patients had a decrease in PLT counts, and the mean level of WBC counts of all SFTS patients was below the normal range, 71.03% (76/107) of patients presented a decrease in WBC counts, overall, 67 patients showed a reduction in neutrophils counts, while lymphocytes count was decreased in 76 patients, in addition, 43.93% of patients displayed a decrease in monocyte counts, while 33 patients demonstrated a reduction in neutrophils counts, lymphocytes and monocyte counts concomitantly. Hb was below the normal range in 23 (21.50%) patients.

**Table 4:** Laboratory results of SFTS patients at admission.

Laboratory test on admission	Normal range	Total (n=107)
**Blood routine**		
WBC (x10^9^/L)	3.50-9.50	2.54 (1.71-4.11)
decreased		76 (71.03%)
Neutrophils counts (x10^9^/L)	1.80-6.30	1.40 (0.94-2.35)
decreased		67 (62.62%)
Lymphocytes counts (x10^9^/L)	1.10-3.20	0.75 (0.47-1.21)
decreased		76 (71.03%)
Monocyte counts (x10^9^/L)	0.10-0.60	0.12 (0.06-0.31)
decreased		47 (43.93%)
Platelet counts (x10^9^/L)	125-350	48.00 (35.50-68.00)
decreased		101 (94.39%)
Hb (g/L)	115-150	128.00 (118.50-141.50)
decreased		23 (21.50%)
**Coagulation function**		
APTT (s)	28-42	51.90 (44.00-60.10)
increased		83 (77.57%)
PT (s)	11.0-16.0	13.73 (1.41)
increased		7 (6.54%)
TT (s)	14.0-21.0	21.70 (18.70-27.90)
increased		54 (50.47%)
D-D (μg/ml)	0.00-0.50	2.70 (1.24-6.35)
increased		84 (78.50%)
FDP (μg/ml)	0.00-5.00	7.72 (3.73-17.92)
increased		57 (53.27%)
**Biochemical markers**		
ALT (u/L)	7-40	78.00 (52.00-123.50)
increased		89 (83.18%)
AST (u/L)	13-35	165.00 (89.50-363.00)
increased		104 (97.20%)
increased		105 (98.13%)
CK (u/L)	40-200	901.96 (1874.39)
increased		70 (65.42%)
TP (g/L)	65.0-85.0	63.67 (6.91)
decreased		69 (64.49%)
Cr (umol/L)	41.0-81.0	71.20 (59.25-91.38)
increased		39 (36.45%)
eGFR (ml/ (min.1.73m^2))	>90	92.50 (69.00-106.00)
decreased		56 (52.34%)
**Infection-related biomarkers**		
CRP (mg/L)	0.00-3.00	3.90 (1.22-12.27)
increased		62 (57.94%)
PCT (ng/ml)	<0.50	0.20 (0.08-0.62)
increased		27 (25.23%)

Markers of coagulation function appeared to be partially disturbed in SFTS patients, The APTT level in 83 (77.57%) patients was above the normal range, and was below the normal range in 5 patients, only 7 patients had a decrease in PT, while 54 patients had a prolonged TT. D-D results were above the normal range in 84 (78.50%) patients. Similarly, FDP was above the normal range in 57 (53.27%) patients. A total of 104 (97.20%) patients had differing degrees of liver function abnormality, with ALT or AST above the normal range; Overall, 69 (64.49%) patients had a decreased TP level, several patients had an abnormal myocardial zymogram, with an elevation in creatine kinase in 70 (65.42%) patients. A proportion of SFTS patients had differing abnormal degrees of kidney function, accompanied by exacerbated Cr levels in 39 (36.45%) patients, while eGFR was below the normal range in 56 (52.34%) patients. The level of common infection-related biomarkers was evaluated, including CRP and PCT, we found an increase of CPR in 62(57.94%) patients while PCT was only increased in 27 (25.23%) patients on admission.

In addition, microbiological testing of SFTS patients was also performed. *Aspergillus fumigatus* was cultured in two patients, and *candida glabrata* was also cultured in one patient, stenotrophomonas maltophilia was detected in one patient along with *klebsiella pneumoniae* infection.

### Comparative Analyses of Variations in Laboratory Results Between Survivors and Non-Survivors

Univariable analysis was conducted between survival and non-survival SFTS patients. The results show that survivors had a higher level of PLT count, TP, and eGFR, meanwhile, levels of APTT, TT, D-D, FDP, ALT, AST, the AST/ALT-ratio, Cr, CK, and PCT were all higher in non-survivors, indicating that advanced age, thrombopenia, prolonged APTT/TT, together with elevated FDP, ALT, AST, AST/ALT-ratio, CK, D-D, Cr, and PCT and decreased TP and eGFR were all associated with SFTS mortality events (**Table 5**).

**Table 5:** Difference of Laboratory results between survivors and non-survivors.

Laboratory test on admission	survivors (81)	non-survivors (26)	p
Age (years)	58.88 ± 11.39	65.08 ± 9.73	0.014
Futime (days)	41.00 (39.00-46.00)	7.00 (3.00-10.00)	<0.001
Hb (g/L)	129.72 ± 20.04	126.15 ± 21.92	0.443
Lymphocytes (x10^9^/L)	0.75 (0.47-1.22)	0.72 (0.52-0.95)	0.755
Monocyte (x10^9^/L)	0.15 (0.07-0.34)	0.10 (0.06-0.15)	0.117
Neutrophils (x10^9^/L)	1.32 (0.94-2.36)	1.54 (1.09-2.13)	0.634
PLT (x10^9^/L)	54.00 (37.00-73.00)	38.50 (23.00-52.50)	0.003
WBC (x10^9^/L)	2.54 (1.67-4.55)	2.62 (1.90-3.54)	0.896
APTT (s)	48.70 (42.77-55.12)	65.40 (57.40-76.20)	<0.001
PT (s)	13.50 (12.80-14.03)	13.60 (12.80-14.90)	0.302
D-D (μg/ml)	2.04 (1.12-4.41)	5.87 (3.84-8.85)	0.003
FDP (μg/ml)	5.75 (3.67-13.38)	17.50 (11.02-31.88)	0.002
TT (s)	20.50 (18.40-23.62)	32.10 (22.70-63.90)	<0.001
eGFR (ml/ (min.1.73m^2))	97.00 (80.00-108.25)	70.50 (59.00-92.00)	<0.001
ALT (u/L)	72.00 (50.00-95.00)	144.00 (69.50-238.00)	0.003
AST (u/L)	123.00 (86.00-231.00)	457.00 (237.50-925.50)	<0.001
AST/ALT-ratio	1.91 (1.47-2.68)	3.14 (2.38-4.22)	<0.001
Cr (umol/L)	67.40 (57.20-86.38)	80.30 (67.95-109.00)	0.017
CK (u/L)	275.00 (119.00-808.00)	586.50 (283.25-1503.25)	0.010
TP (g/L)	64.49 ± 7.23	61.10 ± 5.11	0.029
CRP (mg/L)	3.80 (1.28-10.25)	5.61 (1.28-18.11)	0.245
PCT (ng/ml)	0.16 (0.06-0.36)	0.43 (0.27-1.52)	<0.001

### AST/ALT-Ratio Was an Independent Risk Factor for Prognosis in SFTS Patients

Assignment of variables in Cox regression analysis of laboratory test indicators was shown in **Table 6**. We included all variables into univariate Cox regression analysis (**Table 7**). Results from univariate Cox regression analysis revealed that elevated FDP (HR, 4.13; 95% CI, 1.22-13.95; p =0.022), TT (HR, 4.13; 95% CI, 1.55-11.01; p = 0.004), AST/ALT-ratio (HR, 3.22; 95% CI, 1.356-7.680; p =0.008), and PCT (HR, 2.80; 95% CI, 1.28-6.14; p =0.010), as well as decreased eGFR (HR, 2.94; 95% CI, 1.28-6.76; p =0.011), PLT (HR, 4.66; 95% CI, 1.399-15.533; p =0.012) and manifestation of CNS symptoms (HR, 240; 95% CI, 1.11-5.19; p =0.026) were significant predictors for prognostic of SFTS.

**Table 6:** The independent variables and their assignment incorporated into cox regression analysis.

Laboratory test on admission	value
WBC(x10^9^/L)	<3.5 = 1, ≥3.5 = 0
Neutrophils counts(x109/L)	<1.8 = 1, ≥1.8 = 0
Monocyte counts(x10^9^/L)	<0.1 = 1, ≥0.1 = 0
Lymphocytes counts(x109/L)	<1.1 = 1, ≥1.1 = 0
Platelet counts(x109/L)	<125 = 1, ≥125 = 0
Hb (g/L)	<115 = 1, ≤115 = 0
APTT (s)	>42 = 1, ≤42 = 0
PT (s)	>16 = 1, ≤16 = 0
TT (s)	>21 = 1, ≤21 = 0
D-D (μg/ml)	>0.5 = 1, ≤0.5 = 0
FDP (μg/ml)	>5 = 1, ≤5 = 0
ALT (u/L)	>40 = 1, ≤40 = 0
AST(u/L)	<35 = 0, ≥35 = 1
^*^AST/ALT-ratio	>2.33 = 1, ≤2.33 = 0
CK(u/L)	<200 = 0, ≥200 = 1
TP (g/L)	<65 = 1, ≥65 = 0
Cr (umol/L)	>81 = 1, ≤81 = 0
eGFR (ml/(min.1.73m^2))	<90 = 1, ≥90 = 0
CRP (mg/L)	>3 = 1, ≤3 = 0
PCT (ng/ml)	>0.5 = 1, ≤0.5 = 0

*AST/ALT-ratio was grouped according to the median.

**Table 7:** Univariate cox regression analysis of risk factors for disease prognosis.

Parameters	HR (95%CI)	p
WBC (x109/L)		
0	1.0	0.799
1	1.12 (0.47, 2.66)	
Neutrophils counts(x109/L)		
0	1.0	0.923
1	0.96 (0.44, 2.12)	
Lymphocytes counts(x109/L)		
0	1.0	0.433
1	1.44 (0.58, 3.59)	
Monocyte counts (x109/L)		
0	1.0	0.496
1	1.30 (0.60, 2.82)	
Platelet counts (x109/L)		
0	1.0	0.345
1	4.66 (1.339, 15.33)	
Hb (g/L)		
0	1.0	0.332
1	0.59 (0.20, 1.71)	
APTT (s)		
0	1.0	0.082
1	5.90 (0.80,43.60)	
TT (s)		
0	1.0	0.004
1	4.13 (1.55,11.01)	
D-D (μg/ml)		
0	1.0	0.655
1	1.58 (0.21, 11.74)	
FDP (μg/ml)		
0	1.0	0.022
1	4.13 (1.22, 13.95)	
ALT (u/L)		
0	1.0	0.394
1	1.69 (0.51, 5.62)	
AST (u/L)		
0	1.0	0.752
1	0.72 (0.10, 5.35)	
AST/ALT-ratio		
0	1.0	0.008
1	3.23 (1.35, 7.68)	
CK (u/L)		
0	1.0	0.089
1	2.33 (0.88, 6.18)	
TP (g/L)		
0	1.0	0.161
1	1.92 (0.77, 4.78)	
Cr (umol/L)		
0	1.0	0.130
1	1.81 (0.84, 3.90)	
eGFR (ml/(min.1.73m^2))		
0	1.0	0.011
1	2.94 (1.28, 6.76)	
CRP (mg/L)		
0	1.0	0.660
1	1.20 (0.53, 2.69)	
PCT (ng/ml)		
0	1.0	0.010
1	2.80 (1.28, 6.14)	
Fever		
0	1.0	0.448
1	0.63 (0.19, 2.09)	
Skin change		
0	1.0	0.677
1	1.23 (0.46, 3.26)	
Rigor		
0	1.0	0.338
1	0.69 (0.32, 1.48)	
Fatigue		
0	1.0	0.886
1	0.94 (0.42,2.11)	
Patients with digestive tract symptom		
0	1.0	0.201
1	0.59 (0.27,1.31)	
muscular soreness		
0	1.0	0.457
1	1.34 (0.62, 2.93)	
Patients with CNS symptom		
0	1.0	0.026
1	2.40 (1.11, 5.19)	

Variables with a p-value < 0.1 from univariate Cox regression, including PLT, APTT, FDP, TT, eGFR, AST/ALT-ratio, PCT, CK, and patients with CNS, were subsequently incorporated into a multivariate Cox regression study (**Table 8**), and we found AST/ALT-ratio (HR, 3.38; 95% CI, 1.22-9.30; p =0.018) and PCT (HR, 2.55; 95% CI, 1.11-5.19; p =0.034) were an independent risk factor for prognosis, with no change of HR value for either adjusted age and gender, or age, gender, history of smoking, drinking, surgery, cardiovascular diseases, and diabetes, indicating that AST/ALT-ratio and PCT were a stable risk factor for prognosis in SFTS patients.

**Table 8:** Multivariate cox regression analysis of risk factors for disease prognosis.

	Model1	Model2	Model3
AST/ALT-ratio			
0	1.0	1.0	1.0
1	3.38 (1.22, 9.309) 0.018	3.38 (1.22, 9.30) 0.018	3.38 (1.22, 9.30) 0.018
PCT			
0	1.0	1.0	1.0
1	2.55 (1.07, 6.03) 0.034	2.55 (1.07, 6.03) 0.034	2.55 (1.07, 6.03) 0.034

Model1: unadjusted.

Model2: adjusted age and gender.

Model3: adjusted age, gender, history of smoking, drinking, surgery, cardiovascular diseases, and diabetes.

**Figure 1:**
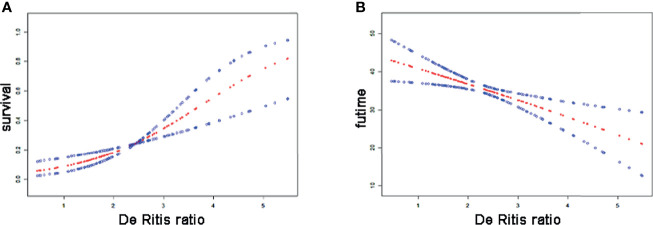
Smoothing splines of De Ritis ratio and risk of mortality or futime of SFTS patients generated in generalized additive models. The solid line and dashed line represent the estimated hazard ratios and their corresponding 95% CIs. General associations between De Ritis ratio and risk of mortality **(A)**, and futime **(B)**.

The results from smooth curve fitting showed a positive linear association between the De Ritis ratio and the risk of mortality in SFTS (**Figure 1A**), and a negative linear association was identified between De Ritis ratio and survival time (**Figure 1B**). Furthermore, univariate Cox regression analysis was also performed using the five complete data points obtained by MI (**Table 9**), variables with p-value less than 0.1 including PLT, FDP, TT, eGFR, AST/ALT-ratio, PCT, CK, and CNS, were incorporated into multivariate Cox regression into the five complete data respectively, AST/ALT-ratio was an independent risk factor for prognosis according to results from the first and fifth data filled by MI (**Table 10**), which was an interesting finding in our research, we further to explore the value of AST/ALT-ratio in SFTS patients.

**Table 9:** Univariate cox regression analysis in five complete data obtained by MI.

	Data = 1	Data = 2	Data = 3	Data = 4	Data = 5	merge
	HR (95%CI)	HR (95%CI)	HR (95%CI)	HR (95%CI)	HR (95%CI)	HR (95%CI)
APTT						
0	1.0	1.0	1.0	1.0	1.0	1.0
1	6.534 (0.885,48.236)	3.846 (0.909,16.276)	7.403 (1.003,54.649)	3.206 (0.758,13.569)	6.118 (0.829,45.157)	5.157 (0.716,37.139)
PT						
0	1.0	1.0	1.0	1.0	1.0	1.0
1	2.434 (0.837,7.078)	2.909 (1.000,8.466)	2.087 (0.718,6.064)	1.205 (0.362,4.013)	1.367 (0.410,4.555)	1.894 (0.451,7.952)
D-D						
0	1.0	1.0	1.0	1.0	1.0	1.0
1	1.462 (0.439,4.870)	1.208 (0.363,4.024)	1.172 (0.352,3.903)	1.063 (0.366,3.085)	3.144 (0.426,23.204)	1.472 (0.263,8.240)
FDP						
0	1.0	1.0	1.0	1.0	1.0	1.0
1	3.018 (1.138,8.008)	2.790 (1.052,7.401)	2.519 (1.011,6.276)	4.022 (1.385,11.679)	5.380 (1.614,17.927)	3.407 (0.967,12.002)
TT						
0	1.0	1.0	1.0	1.0	1.0	1.0
1	3.246 (1.303,8.087)	3.689 (1.481,9.191)	4.585 (1.728,12.166)	4.209 (1.586,11.170)	3.388 (1.360,8.439)	3.791 (1.406,10.223)
eGFR						
0	1.0	1.0	1.0	1.0	1.0	1.0
1	2.866 (1.245,6.597)	2.866 (1.245,6.597)	2.866 (1.245,6.597)	2.993 (1.300,6.891)	2.993 (1.300,6.891)	2.916 (1.264,6.724)
CK						
0	1.0	1.0	1.0	1.0	1.0	1.0
1	2.252 (0.849,5.974)	2.470 (0.931,6.551)	2.359 (0.889,6.258)	2.470 (0.931,6.551)	2.470 (0.931,6.551)	2.402 (0.902,6.398)
CRP						
0	1.0	1.0	1.0	1.0	1.0	1.0
1	1.201 (0.536,2.695)	1.201 (0.536,2.695)	1.256 (0.560,2.818)	1.256 (0.560,2.818)	1.201 (0.536,2.695)	1.223 (0.544,2.748)
PCT						
0	1.0	1.0	1.0	1.0	1.0	1.0
1	2.436 (1.126,5.269)	2.920 (1.352,6.305)	2.617 (1.212,5.650)	2.729 (1.261,5.906)	2.617 (1.212,5.650)	2.659 (1.214,5.823)

**Table 10:** Multivariate cox regression analysis in five complete data obtained by MI.

	Variables	HR (95%CI)	p
Data=1	PLT	3.299 (0.964,11.287)	0.057
	AST/ALT-ratio	2.503 (1.035,6.054)	0.042
	PCT	2.162 (0.996,4.693)	0.051
Data=2	TT	3.689 (1.481,9.191)	0.005
Data=3	TT	4.585 (1.728,12.166)	0.002
Data=4	TT	4.209 (1.586,11.170)	0.004
Data=5	AST/ALT-ratio	2.429 (1.006,5.865)	0.049
	PCT	2.458 (1.137,5.316)	0.022
	FDP	4.052 (1.191,13.783)	0.025

**Figure 2:**
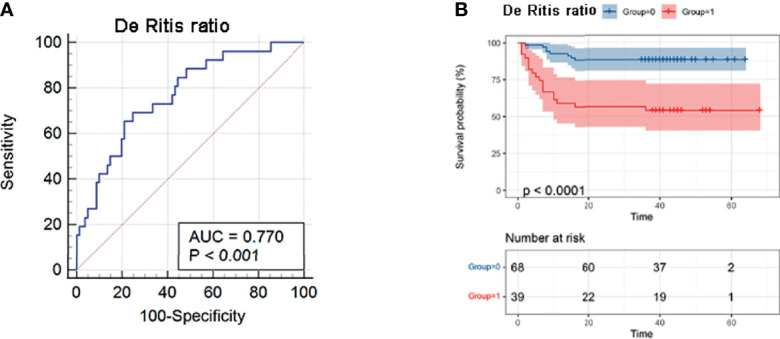
Value of AST/ALT-ratio in predicting prognosis of SFTS patients. **(A)** Receiver operating curve (ROC)-analysis. The area under the curve (AUC) for survival regarding the AST/ALT-ratio was 0.770 (95%CI, 0.679-0.846). **(B)** Kaplan-Meier curves predicting survival, grouped by the cut-off value of AST/ALT-ratio.

The ideal cut-off value of AST/ALT-ratio in predicting prognosis was determined through ROC analysis (**Figure 2A**), according to the ROC curve, the optimal cut-off value was 2.683, with a sensitivity of 0.692 and a specificity of 0.753 (AUC=0.770, 95%CI 0.679-0.846), and Kaplan–Meier survival analysis showed that SFTS patients with AST/ALT-ratio >2.683 were associated with a reduced futime, therefore indicating an unfavorable prognosis (**Figure 2B**).

### Validation of Model Accuracy

To confirm the results of multivariate Cox analysis, the PH assumption of classification variables (PLT, FDP, TT, eGFR, AST/ALT-ratio, PCT, CK, and CNS) were determined (**Figure S1A**), the survival curves of each group of classification variables were basically parallel or equidistant, suggesting the PH assumption could be determined to be satisfied, indicating a credible result of our analysis. Besides, the calibration curve also showed a good predictive value of our model (**Figure S1B**), furthermore, our proposed model demonstrated good discriminative ability with a C statistic of 0.68 for predicting OS, result from Hosmer and Lemeshow Test (H-L Test) (p=0.985) indicated a predictive accuracy of AST/ALT-ratio. Collectively, these data strongly suggested our analysis may have a good prediction value.

### Pairwise Correlation Between the De Ritis Ratio and Clinical Parameters

**Figure 3:**
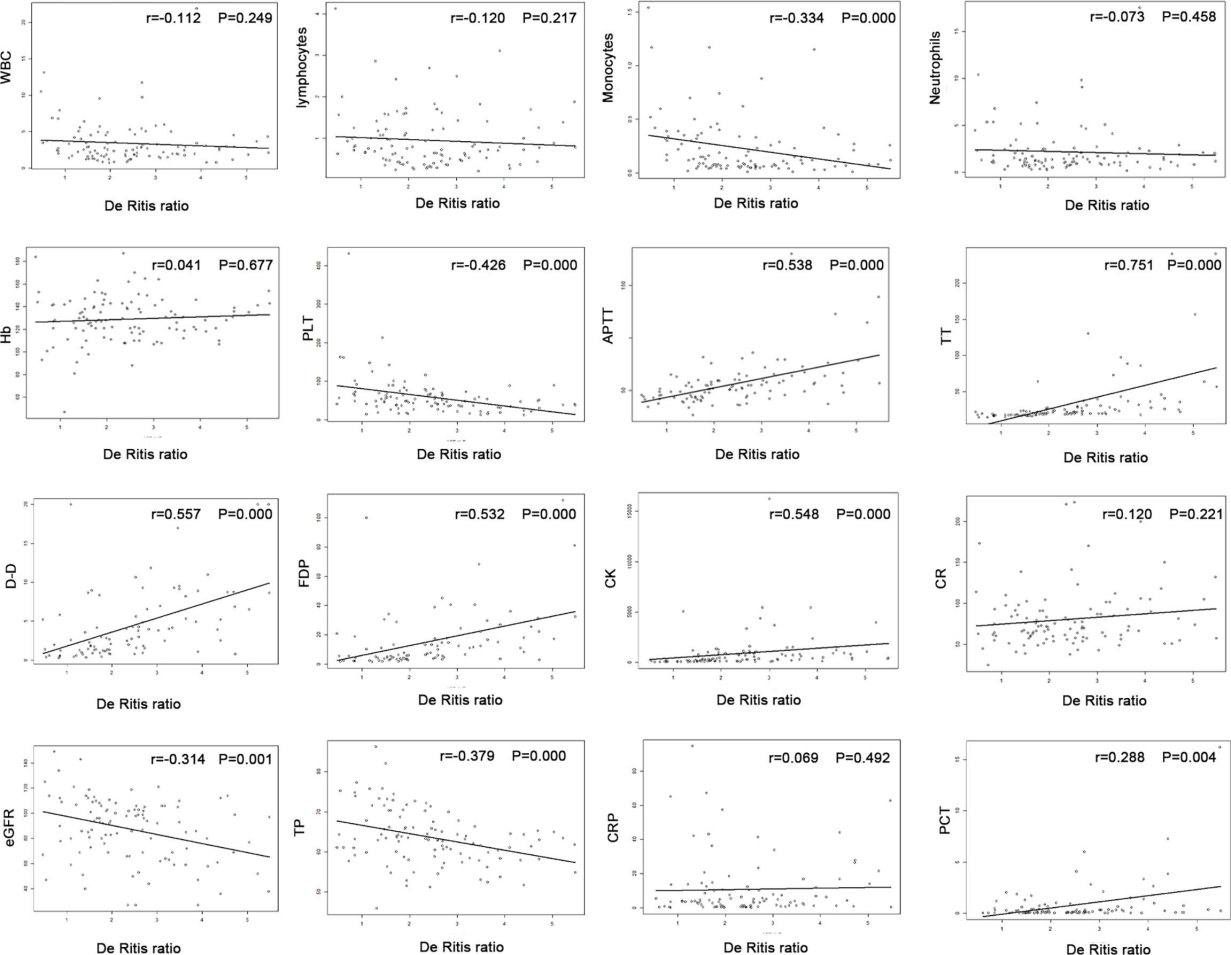
Pairwise correlation between the De Ritis ratio and clinical parameters.

In our research, we found De Ritis ratio showed a strong positive correlation with APTT (r=0.538, P<0.001), D-D (r=0.557, P<0.001), FDP (r=0.532, P<0.001), TT (r=0.751, P<0.001), CK (r=0.548, P<0.001), and PCT (r=0.288, P=0.004), while inversely associated with PLT (r =-0.426; P <0:001), monocyte (r =-0.3337; P =0.004), eGFR (r =-0.3137; P =0.001), and TP (r =-0.379; P <0:001) (**Figure 3**).

### Shifting Trend of De Ritis Ratio and PLT of SFTS Patients Treated With Ribavirin

**Figure 4:**
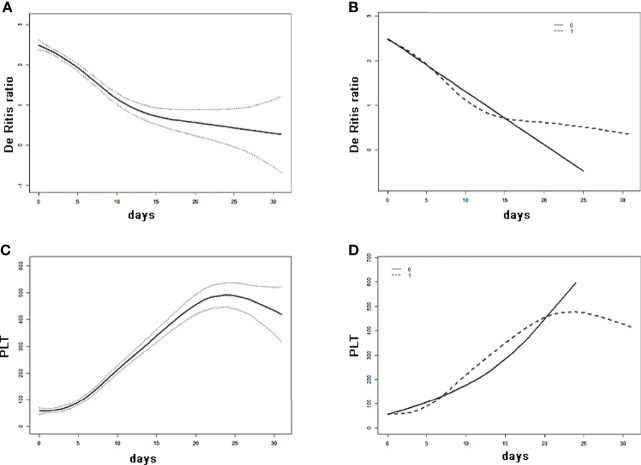
Shift trend of De Ritis ratio and PLT of SFTS patients analyzed by GAMM. **(A)** The overall change of De Ritis ratio in 107 SFTS patients. **(B)** The change of De Ritis ratio in SFTS patients treated/untreated with ribavirin. **(C)** The overall change in PLT within 107 SFTS patients. **(D)** The change of PLT in SFTS patients treated/untreated with ribavirin.

A total of 89 patients received ribavirin for treatment among the 107 SFTS patients, we divided patients into two groups according to different treatment strategies, continuous changes in AST/ALT-ratio, and PLT in SFTS patients undergoing differing GAMM treatments were analyzed (**Figure 4**). Evidently, the variation trend of the AST/ALT-ratio in all 107 SFTS patients initially decreased remarkably and then shifted to gradual decline, while PLT increased during treatment and in patients treated with ribavirin. Shift trends for the De Ritis ratio corresponded with the overall trend, although a plummet was observed in patients not treated with ribavirin, and the magnitude of PLT increase in patients receiving ribavirin was higher.

## Discussion

SFTS is an emerging infectious disease with high mortality. This descriptive study focused on the epidemiology and clinical characteristics of SFTS, including data on 107 patients infected with SFTSV.

SFTSV is confirmed to be mainly transmitted by ticks (arthropod-borne infection). However, direct contact with STSF patient blood was also significantly associated with SFTS infection (Tang et al., 2013), and nosocomial person-to-person transmission of SFTS has been reported, suggesting a human-to-human transmission of the disease (Jung et al., 2019). In our collected cases, one SFTS case was medical staff, that came into direct contact with infected blood samples. In addition, three patients were infected because of intimate contact with infected patients, verifying the human-to-human transmission of SFTSV, rendering it important to prevent SFTSV transmission through early diagnosis of SFTS and overall cautionary behavior.

Clinical features of SFTS were non-specific, a sudden onset of pyrexia (temperature 38–41°C) was the initial hallmark symptom manifestation. This study cohort highlighted 59.81% of patients to have a temperature > 38.6°C on admission, SFTS also caused rigor in patients, with over 33% of investigated patients having muscular soreness, most patients felt fatigued, indicating a muscle tissue invasion of SFTSV. Being a viral infection, gastrointestinal/neurological symptoms were commonplace. Most patients had a good outcome, while a few patients progressed into multiple organ failure (MOF), which was a major risk factor for mortality events. The capacity of SFTSV replication and the status of host immune response affected the severity and clinical outcome of patients. Skin changes appeared in a proportion of patients in our survey, one research found that SFTSV RNA was detected in histopathological skin biopsy specimens and detected virus-infected cells dermal cells from such patients, though the mechanism for this finding needs to be clarified further (Sato et al., 2019). A co-infection of fungi was found in several severe patients in our cases, possibly caused due to suppressed cellular immune function in SFTS patients, a marked fungal infection was also observed in the lungs of SFTS patients in Japan (Hiraki et al., 2014), suggesting that further focus is required for fungal infections during SFTS progression.

In terms of laboratory results of SFTS patients at admission, the absolute value of platelets and leukocytes were decreased within most patients, however, the mechanism of this shift was not well described, a study revealed that thrombocytopenia could be associated with arginine deficiency in SFTS patients (Li et al., 2018). Conflicting opinions stated that SFTSV-induced thrombocytopenia could be due to viral adhesion to platelets, triggering splenic macrophages to recognize and phagocytose platelets (Jin et al., 2012). Researchers established an SFTSV-infection cell line model and determined the infectivity and stimulation of SFTSV on vascular endothelial cells *in vitro*, revealing a vascular endothelial cell injury and barrier function damage caused by SFTSV (Li et al., 2018; Westover et al., 2019). Moreover, we found an overall reduction in the absolute value of lymphocytes and neutrophils, with a proportion of patients exhibiting a reduction in monocyte counts, consistent with multiple studies (Peng et al., 2016), furthermore, monocytic apoptosis in the early stage of infection thwarted the differentiation and maturation of lymphocytes (Song et al., 2018).

Coagulation disturbances were observed in most patients, especially prolonged APTT, indicating issues within the endogenous coagulation pathway, D-D and FDP, degradation products of fibrin, were exacerbated in most patients, suggesting a hypercoagulable state and secondary hyperfibrinolysis and consequently leading to disseminated intravascular coagulation (Zhang et al., 2012), became significant risk factor for death of SFTS patients. AST, ALT, and CK were increased in more than 75% of patients, meanwhile, more than half of patients had a decrease in TP, indicating hepatic injury on SFTSV infection. Some patients progressed into renal damage, as demonstrated in this study, the Cr was also decreased and eGFR had decline in some cases.

An important finding was discovered during analysis of SFTS risk factors, where the AST/ALT-ratio was an independent risk factor for prognosis and, to the best of our knowledge, our study was the first to evaluate the potential prognostic value of AST/ALT-ratio in SFTS, patients with AST/ALT-ratio >2.683 was associated with a shorter futime, De Ritis ratio affects the SFTS mortality strongly, and this influence persisted after adjusting for variables. Furthermore, we also found a new prospective relationship between the De Ritis ratio and different clinical parameters for SFTS, De Ritis ratio was inversely associated with PLT, previous studies highlighted that platelets are essential cells that contribute to cellular inflammation and are used in the scoring system for hepatic fibrosis. Megan G. Hofmeister et al. found that an elevated De Ritis ratio and low platelet counts were associated with higher odds of hepatitis A-related mortality events (Hofmeister et al., 2020), moreover, low platelet counts were also associated with cirrhosis and progression to hepatic decompensation among patients with chronic hepatitis C (Xu et al., 2016). SFTS patients with a high De Ritis ratio and decreased platelet counts may indicate a progression of liver injury. De Ritis ratio was also inversely related to the level of eGFR and TP, meanwhile, a positive relationship with the coagulation function (APTT, D-D, FDP, and TT) was also denoted. Interestingly, serum AST/ALT-ratio levels were previously described as a biomarker for hepatitis B viral activity in the liver and, consequently, a poor prognosis in patients with hepatocellular carcinoma (Poon et al., 2000) was identified, the De Ritis ratio was also related to a high mortality rate in multiple cancer models in other previous studies. An AST/ALT ratio > 1.44 was an independent prognostic factor for poor cancer-specific survival and OS in patients with Oral and oropharyngeal cancer (Knittelfelder et al., 2020), De Ritis emerged as a valid prognostic marker for disease-free survival (DFS) in stage 2 and stage 3 non-metastatic colorectal cancer patients (Scheipner et al., 2021). It also predicts the prognosis of patients with gastric (Li et al., 2020), prostate (Zhou et al., 2020), advanced-stage pancreatic cancer (Varol et al., 2020), furthermore, it is considered to be a novel and inexpensive marker for individual risk assessment in the treatment of pancreatic cancer (Riedl et al., 2020). In general, De Ritis is widely expressed in differing tissues, however, the underlying pathophysiologic mechanism of elevated De Ritis ratio and its prognostic value in SFTS patients is still unclear. Cytokine storm-mediated immune activation and mechanisms of impairment of innate immune responses could justify the main pathogenesis of SFTS virus infection in recent research. A study conducted by Sun and colleagues (Sun et al., 2015) found a susceptibility of human hepatic epithelial cells to SFTSV, and in HepG2 cells, TNF-α and FasL were induced robustly up to 36- and 21-fold in 72h after infection of SFTSV, this latter finding indicates an extrinsic apoptotic pathway was fully activated in HepG2 by NF-KB signal pathway, moreover, pro-inflammatory cytokines including serum IL-6 and TNF-α dramatically increased as the severity of the disease increases, while inhibition of TLR3 expression was peripheral mDCs and monocytes in SFTS deceased cases (Song et al., 2017). Regarding patients with allogeneic hematopoietic stem cell transplantation, such a cohort presented increments in TNF-α, IFN-γ, and IL-2 levels, accompanied with an elevation of ALT and AST (Lv et al., 2017), this could indicate a release of cytokines that possibly activates T cells to mount immune responses on target organs, leading to an increase in De Ritis-ratio within SFTS. However, the exact mechanism needs to be explored further.

Findings pointed out that ribavirin had inhibitory activity against SFTSV replication *in vitro (* Lee et al., 2017), there have also been reports that ribavirin had a minimal impact to alleviate disease progression of SFTS syndrome (Shimojima et al., 2015). In our research, we found that ribavirin deployment serum reduced AST levels more drastically, in comparison to non-treated cases, while PLT was distinctly increased, and the PLT reverted to normal levels post-antiviral treatment. However, Liu found that receipt of ribavirin therapy had no significant effect on neither increased platelet counts nor viral loads during hospitalization of patients with fatal or non-fatal cases (Lv et al., 2017), conversely to the findings of this study. Consequently, further studies are required on this research niche.

In essence, measurement of AST/ALT-ratio is a minimally invasive procedure and a simple, inexpensive biomarker that shows potential to distinguish patients who are at high risk and should be prospectively evaluated as a potential selection criterion for risk factor-stratified management in SFTS patients and for adjuvant treatment trials.

Our study had several important limitations. Firstly, a small sample size and a deficiency of external data validation were the biggest flaws of our study, this reduced sample size limits the validity of our findings, while the retrospective and single-center study could have also added bias to our results. Secondly, details of ribavirin treatment regimens were not included in this study, and a specific criterion was not set to evaluate the curative effect, another limitation was the viral load was not included, since only qualitative analysis was conducted in our hospital. Such drawbacks will be addressed in our future studies.

## Conclusion

This study provided detailed clinical characteristics and laboratory data of patients infected with SFTSV, symptoms of SFTS were non-specific, such as fever and thrombocytopenia, infection of SFTSV could lead to a multiple organ injury. AST/ALT-ratio was an independent risk factor for prognosis, though the specific mechanism for such a finding requires further studies.

## Data Availability Statement

The original contributions presented in the study are included in the article/**Supplementary Material**. Further inquiries can be directed to the corresponding authors.

## Ethics Statement

The studies involving human participants were reviewed and approved by Medical Ethics Committee of the First Affiliated Hospital of Anhui Medical University with the following reference number: Quick-PJ 2021-06-18. Written informed consent to participate in this study was provided by the participants’ legal guardian/next of kin.

## Author Contributions

LW, TL, and YHX were involved in the study design and writing of the article. YX participated in the data collection. SZ and AB were involved in the data analysis. All authors reviewed and approved the final manuscript.

## Conflict of Interest

The authors declare that the research was conducted in the absence of any commercial or financial relationships that could be construed as a potential conflict of interest.

## Publisher’s Note

All claims expressed in this article are solely those of the authors and do not necessarily represent those of their affiliated organizations, or those of the publisher, the editors and the reviewers. Any product that may be evaluated in this article, or claim that may be made by its manufacturer, is not guaranteed or endorsed by the publisher.
