# Supplementary figures and images for: The AST/ALT Ratio (De Ritis Ratio) Represents an Unfavorable Prognosis in Patients in Early-Stage SFTS: An Observational Cohort Study
Source: Front Cell Infect Microbiol. 2022 Feb 8;12:725642. doi: 10.3389/fcimb.2022.725642 (PMC8861437; doi:10.3389/fcimb.2022.725642)

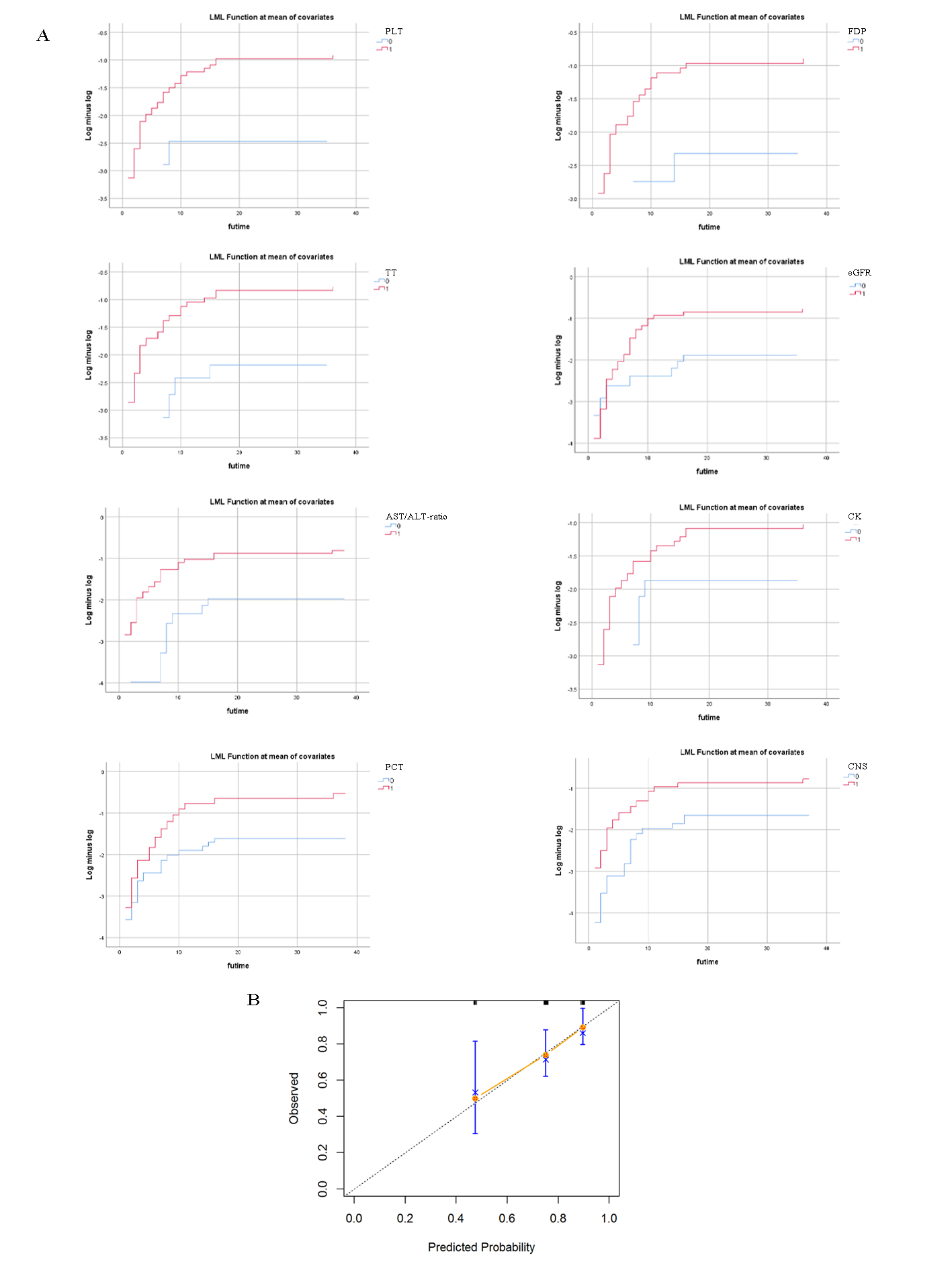

Supplement: Supplementary file 1 [file Image_1.tif]
